# Medicinal Plants and Other Living Organisms with Antitumor Potential against Lung Cancer

**DOI:** 10.1155/2014/604152

**Published:** 2014-07-24

**Authors:** Luara de Sousa Monteiro, Katherine Xavier Bastos, José Maria Barbosa-Filho, Petrônio Filgueiras de Athayde-Filho, Margareth de Fátima Formiga Melo Diniz, Marianna Vieira Sobral

**Affiliations:** Department of Pharmaceutical Sciences, Federal University of Paraiba, 58051-900 João Pessoa, PB, Brazil

## Abstract

Lung cancer is a disease with high morbidity and mortality rates. As a result, it is often associated with a significant amount of suffering and a general decrease in the quality of life. Herbal medicines are recognized as an attractive approach to lung cancer therapy with little side effects and are a major source of new drugs. The aim of this work was to review the medicinal plants and other living organisms with antitumor potential against lung cancer. The assays were conducted with animals and humans, and Lewis lung carcinoma was the most used experimental model. China, Japan, South Korea, and Ethiopia were the countries that most published studies of species with antitumor activity. Of the 38 plants evaluated, 27 demonstrated antitumor activity. In addition, six other living organisms were cited for antitumor activity against lung cancer. Mechanisms of action, combination with chemotherapeutic drugs, and new technologies to increase activity and reduce the toxicity of the treatment are discussed. This review was based on the NAPRALERT databank, Web of Science, and Chemical Abstracts. This work shows that natural products from plants continue to be a rich source of herbal medicines or biologically active compounds against cancer.

## 1. Introduction

Cancer is a collection of heterogeneous genetic diseases united by common alterations in multiple cellular signaling pathways [1]. Various hallmarks have been proposed for cancer cells. Evasion of programmed cell death or apoptosis has been recognized as one of the main alterations that dictate malignant growth [2]. Furthermore, other features include self-sufficiency in growth signaling, deregulation of cellular energetics, sustained angiogenesis, evasion of immune detection, and tissue invasion and metastasis [1–3]. In addition, two characteristics of cancer that facilitate acquisition of these hallmarks have been described: genome instability and mutation tumor-promoting inflammation [2]. Coordinated processes such as cell proliferation, differentiation, and apoptosis are modified, producing altered cellular phenotypes with these specific characteristics [1, 2].

According to the World Health Organization (WHO), cancer represents noncommunicable diseases responsible for 63% of deaths worldwide, where it is characterized as the second cause of death in western countries [4, 5]. Its incidence is strongly affected by demographic aspects, such as population aging, eating habits, and, in particular, environmental factors such as exposure to ultraviolet rays. The International Agency for Research on Cancer (IARC) estimates that there were 12.7 million new cancer cases in 2008 and that this number is expected to grow to 21.4 million by 2030 [6, 7].

Lung cancer is currently the malignant tumor with the highest mortality rate worldwide, often because it is not detected until there has been substantial progression of the illness, which leads to a significant reduction in quality of life of the patient [8]. Different factors are pointed out as possible causes of lung cancer, including active cigarette smoking, exposure to secondhand cigarette smoke (passive smoking), pipe and cigar smoking, exposure to indoor and outdoor air pollution, exposure to radiation, and occupational exposure to agents such as asbestos, nickel, chromium, and arsenic [9]. The most important risk factor is smoking [10] and the incidence rates of lung cancer are generally higher among men than women [11]. Lung cancer is classified into nonsmall cell lung carcinoma (NSCLC), including squamous cell carcinoma, adenocarcinoma, and large cell carcinoma, which represents 80% of all lung cancer cases, and the remaining cases are small cell lung carcinoma (SCLC) [11, 12]. Recently, a new classification was published that defined “molecular subtypes” of lung cancer based on specific actionable genetic aberrations [13]. Many strategies have been studied for lung cancer chemoprevention and treatment, including STAT3 pathway inhibition (e.g., curcumin), cell-cycle arrest (e.g., gambogenic acid), hTERT silencing, miRNA modulation, iNOS suppression, chitin inhibition, angiotensin receptor blockade, TGF-*β* antagonism, VEGFR-2/EGFR inhibition (e.g., vandetanib), and Nrf2 activation [14]. Some strategies have been proven effective in human trials, for example, the use of ceritinib, a new ALK (anaplastic lymphoma kinase gene) inhibitor, in patients with advanced lung cancers harboring genetic alterations in ALK, in a phase 1 study [15]. In addition, the study of tumor-associated biomarkers has grown with the aim of reducing the mortality rate of lung cancer by early diagnosis and prognosis [16]. Another approach that has been studied is the use of the cancer stem cell model, which offers new insights into the limitations of current cancer treatments [17]. New opportunities for targeted therapy are under development based on the discovery of multiple molecular mechanisms underlying the development, progression, and prognosis of lung cancer. Among them, the targeted inhibition of the vascular endothelial growth factor (VEGF) or epidermal growth factor (EGF) signaling pathways has been clinically validated in the treatment of advanced NSCLC [18].

Nowadays, surgery, chemotherapy, radiation, hormones, and immunotherapy are the main approaches for the cancer treatment, often supplemented by other complementary and alternative therapies, such as herbal medicines. Although chemotherapy is the method most used, several problems are associated with its use, including limited efficacy, severe toxicity, and multidrug resistance [19].

Plants have a long history of use in the treatment of cancer and continue to be a major source of new drugs [20]. Herbal medicines have been recognized as one of the attractive approaches for lung cancer therapy because they have proven to be useful and effective in sensitizing conventional agents, prolonging patient survival time, preventing side effects of chemotherapy, and improving quality of life in lung cancer patients [21]. A recent cohort study with 453 cancer patients revealed that the percentage of patients using herbal medicines in combination with conventional chemotherapy was approximately 77% [22]. In these cases, natural products as complementary therapy for the treatment of lung cancer were used mainly with the aim of reducing toxicity, alleviating cancer-related symptoms, stimulating the immune system, and even having direct anticancer effects [23].

Many natural products or synthetic analogs are still widely used clinically, for example, the so-called vinca alkaloids, vinblastine and vincristine, isolated from the Madagascar periwinkle,* Catharanthus roseus*, paclitaxel (Taxol), obtained from the leaves of various* Taxus* species, and the two clinically active agents, etoposide and teniposide, which are semisynthetic derivatives of the natural product epipodophyllotoxin [20]. Other promising agents, natural or synthetic analogs, are in the clinical development phase, including flavopiridol [24] and combretastatin A4 [25]. In addition, many compounds obtained from plants or other living organisms, alone or in combination, are still studied by* in vitro* models for determining their mechanisms of action against lung cancer cells [26–37].

The most common forms of therapeutic use of plants worldwide are teas and herbal infusions. Considering that their use has increased in recent years aiming to reduce the risks of cardiovascular disease and cancers, research is going on to characterize this effect. Recent data showed that different teas and herbal infusions widely consumed in Hong Kong, Macau, Taiwan, Mainland China, and many other places in the world have antiproliferative effects against human lung cancer cells [38]. Many cancer patients take these medicines, but their effects at the cellular level are largely unknown. However, different preclinical and clinical studies have shown the antitumor activity of herbal extracts against lung cancer [39]. The data have shown that induction of apoptosis is the major mechanism of action of these extracts. The majority of the articles show the effect of Chinese herbal extracts. Some of them show that the response of lung tumor cells to these extracts was similar to their response to conventional chemotherapeutic drugs, and that their mechanism of action was associated with apoptosis induction [40]. Some examples of medicinal herbs with antitumor activity against lung cancer cells can be cited.* Selaginella tamariscina *is a traditional Chinese herb with antimetastatic effects* in vitro* and* in vivo* against lung cancer cells [41].* Crocus sativus *L. (Saffron) aqueous extract is widely used as a food additive and in traditional medicine for cancer. Its effects against lung cancer cells were investigated, and antilung cancer activity was associated with induction of apoptosis [31]. The bioactive fraction of* Toona sinensis* leaves inhibited H441 xenograft tumor growth in both therapeutic and preventive experiments. An* in vitro* study revealed that the natural product acts by inducing apoptosis [42]. The methanolic fraction of* Sesbania grandiflora *was found to exert potent antiproliferative effects especially against human lung cancer cell lines. This effect was related to the induction of apoptosis associated with high levels of reactive oxygen species (ROS) intermediates [43]. A study of the combination of active components of* Prunella* revealed that total triterpenes and total phenols had antilung cancer activity and that their combination significantly enhanced the activity. Thus, its efficacy against lung cancer was attributable to multiple components acting at an optimal ratio [44].* Descurainia sophia *has been traditionally used in Korean medicine. Recent data from the study of gene expression profile found the antitumor effect of the ethanol extract of the seeds of* D. sophia* against lung cancer to be involved with the regulation of metabolism- and signaling-related pathways [45]. Mountain ginseng butanol extract inhibited lung cancer cell growth by inducing apoptosis. Its mechanisms were associated with a reduction in NF-*κ*B activity and increase in p53 activity [46].

Substances isolated from various medicinal plants have also been extensively studied for their antilung cancer activity. [6]-Gingerol, a pungent ingredient of ginger (*Zingiber officinale*) showed antiangiogenic activity* in vitro* and* in vivo *and reduced the number of lung metastases in mice receiving B16F10 melanoma cells [47]. Embelin, an active component of fruits of* Embelia ribes*, has been demonstrated to possess a broad spectrum of therapeutic properties, including antitumor activity against lung cancer cells. Recent data indicate the crucial role of p38 and JNK pathways in embelin-induced apoptosis [48]. Danshen (*Salvia miltiorrhiza *Bunge) is a traditional medicine that has been used in China to treat various diseases, including cancer. Tanshinone I is a diterpene isolated from this species, which reduced lung adenocarcinoma tumor growth. The studies of its mechanisms showed that the compound inhibits pulmonary tumor formation in an animal model by downregulation of the cell cycle at S and G2/M phases [27]. A carbazole alkaloid isolated from* Murraya koenigii *Spreng, girinimbine, inhibited lung cancer cell growth by inducing both intrinsic and extrinsic pathways of apoptosis [49]. Capilliposide, extracted from a traditional Chinese medicine,* Lysimachia capillipes*, induced apoptosis in NSCLC cells. Xenograft tumor growth was significantly decreased after* in vivo* treatment. The* in vitro* data revealed that capilliposide increased the intracellular level of ROS, which activated the mitochondrial apoptotic pathway [50].* Scutellaria baicalensis* root is used in China as an adjuvant in the chemotherapy of lung cancer. Recently, three compounds were associated with its activity, baicalin, baicalein, and wogonin [51]. Subamolide A, isolated from* Cinnamomum subavenium,* induced lung cancer cell death by ROS generation, which triggers mitotic catastrophe followed by apoptosis [52]. Terpinen-4-ol, a monoterpene component of the essential oils of several aromatic plants, showed its antitumor effect on NSCLC cells by induction of apoptosis, with involvement of the mitochondrial apoptotic pathway [53]. Davallic acid is the major active compound of* Davallia divaricata*, a species traditionally employed in folk medicine for the therapy of lung cancer in Taiwan. Recent data showed that this compound induced oxidative stress and apoptosis in lung cancer cells [54]. Total flavonoids of* Daphne genkwa* inhibited tumor growth and metastasis by protecting the viability of host immunocytes and their proliferation potential and selectively inhibiting tumor cell proliferation. In this fraction, daphnodorin B was the predominant constituent [55].

In addition, many studies have shown the use of combined therapy of herbal medicines or natural substances with chemotherapeutic drugs to enhance their antitumor effects and/or reduce their toxicity. The combination of Xiao-Ai-Ping, a traditional Chinese medicine, and cisplatin promotes the infiltration and function of CD8^+^ T cells and thus enhances the growth-inhibitory effects of cisplatin on LLC xenografts [56].* Marsdenia tenacissima *has long been used as a remedy to treat cancer in China. Its combination with gefitinib, an orally active tyrosine kinase inhibitor, improved gefitinib efficacy in NSCLC regardless of EGFR status [57, 58]. The combined therapy of plants extracts,* Phyllanthus emblica* and* Terminalia bellerica*, with doxorubicin or cisplatin resulted in a synergistic effect and the possibility of reducing the doses of the chemotherapeutic drugs [59]. Fuzheng Fangai pill (FZFA), a traditional Chinese formula, is widely used for cancer treatment. Recent studies showed that FZFA combined with cyclophosphamide (CTX) strongly reduced the growth and metastatic rate of LLC through inhibition of the SOCS/JAK-STAT pathway and inflammatory cytokine responses. In addition, it was observed that the combination had greater activity than CTX alone [60]. Osthole is a natural compound extracted from a number of medicinal plants. The combination of osthole and cisplatin, one of the most active chemotherapeutic agents in lung cancer treatment, resulted in greater efficacy in growth inhibition and apoptosis induction [61]. Meta-analysis of randomized trials showed that* Astragalus*-based Chinese herbal medicine may increase the effectiveness of platinum-based chemotherapy when combined with chemotherapy [62]. A clinical study showed that the combination of Chinese drugs and conventional chemotherapy in NSCLC patients can enhance short-term therapeutic efficacy in the treatment of NSCLC and prolong patients' median survival time, but it did not find any evident impact on median time to progression [63].

New technologies are in development to enhance the activity and reduce the toxicity of natural products during the research of new therapies. Liposomes may be a promising delivery system for drugs in cancer treatment, including lung cancer [64]. The development of liposome-based cisplatin drug called Lipoplatin was beneficial in reducing the nephrotoxicity of cisplatin, the drug of choice for the treatment of NSCLC. Lipoplatin is anticipated to complete phase III clinical trial testing in 2013 and 2014 [65]. *β*-elemene, a sesquiterpene vinyl monomer isolated first from* Curcuma wenyujin* (Zingiberaceae) rhizome, is present in Chinese herbs and plants. Different studies with this compound have shown efficient antilung cancer activity including inhibiting angiogenesis, inducing tumor cell apoptosis, enhancing radiosensitivity, and favorable chemotherapeutic effects in combination with other anticancer agents. In addition,  *β*-elemene has only slight side effects. However, the clinical application of *β*-elemene was limited by its hydrophobic property, poor stability, and low bioavailability. Phase II clinical trials with *β*-elemene are in development, and aiming to improve of its pharmacokinetics new delivery systems are being produced, including liposome-based delivery systems of *β*-elemene. The authors emphasize that *β*-elemene liposomes will be critical for future clinical applications of *β*-elemene in lung cancer treatment [66]. Herbal extracts have also been studied using new technologies. The ethanolic extract of* Polygala senega *(EEPS) caused apoptosis in lung cancer cell line A549. Its poly-(lactic-co-glycolic) acid (PLGA) nanoparticle-encapsulated form enhanced cellular entry and bioavailability and inhibited the growth of lung cancer cells better than EEPS. The apoptosis of A549 cells was associated with decreased expression of survivin and PCNA mRNA and increased expression of caspase-3 and p53 mRNAs of A549 cells. The authors showed that the formulation of EEPS-loaded PLGA nanoparticles was more effective than EEPS, probably due to more aqueous dispersion after nanoencapsulation, and concluded that the nanoencapsulated ethanolic root extract of* P. senega *may serve as a potential chemopreventive agent against lung cancer [67].

In the course of our continuing search for bioactive natural plant products, we have published reviews on crude plant extracts and plant-derived compounds with potential uses [68–77]. Moreover, our group has also reviewed the medicinal and poisonous plants of Northeast Brazil [78], among others [79–84].

The search was carried out in the Web of Science, Chemical Abstracts, and NAPRALERT (acronym for the University of Illinois Natural Products ALERT service) databanks. The references found in the searches were later consulted. For details on the mechanism-based bioassays utilized for antitumor activity, the original references should be consulted.

## 2. Results and Discussion

Natural products have been increasingly used worldwide to treat various diseases, including cancer. Herbal medicines and phytochemicals can be potent agents for lung cancer chemoprevention and treatment by regulating multimolecular targets involved in angiogenesis, metastasis, and severe side effects; only provided quality control and reproducibility issues are solved [21]. Compared with the conventional drugs used in cancer treatment, the toxicity of medicinal plants may seem trivial; however, it is a serious public health problem. Several medicinal plants are considered toxic and can cause serious damage to the health of patients. Therefore, the assessment of the toxicity of medicinal plants, as well as their herbal preparations, is essential to determine the applicability of the sample as a pharmacological drug [85].

In the current review, we present medicinal plants, distributed in diverse parts of the world, with antitumor activity against different* in vivo* lung cancer models (Table 1). The effectiveness of the medicinal plants depended on the type of drug studied and the bioassay model. Thus, it was possible to classify the extracts as active or inactive, and the mechanisms of action studied were discussed.

Celastraceae was the most cited family for antitumor activity in lung cancer models, followed by Araliaceae, Euphorbiaceae, and Fabaceae.* Maytenus serrata* was the only species studied of the family Celastraceae, showing antitumor activity for its fruit, root, and stem wood.* Eleutherococcus senticosus* and* Panax ginseng* red type were the evaluated species of the family Araliaceae. Among the four mentioned for* P. ginseng* red type, one of the studies showed the antitumor activity of this species against lung adenoma induced by different carcinogenic agents. Studies with* P. ginseng* were carried out with the ethanol-insoluble fraction, obtained from the fractionated water extract. The tumor was induced by a single subcutaneous (*s.c*.) injection of benzo[*α*]pyrene (BP), and the treatment with* P. ginseng* significantly inhibited lung tumor incidence. The investigation of the mechanisms of action showed proliferation of splenocytes and generation of activated killer cells* in vitro*, suggesting immunomodulatory effects [86].

*Euphorbia esula*,* Euphorbia fischeriana*,* Euphorbia ingens,* and* Croton macrostachys *were the most studied Euphorbiaceae species. All of them showed significant antitumor activity against lung cancer models. Crotepoxide, a novel cyclohexane diepoxide obtained from* C. macrostachys* and alcoholic extracts of the fruits of this species, inhibited the growth of LLC in mice [87].

Among the active species of the family Fabaceae,* Sophora flavescens* and* Cassia garrettiana* were cited. The methanol extract of* C. garrettiana* was found to contain the substances identified as cassigarol A and piceatannol. The extract and isolated substances inhibited tumor growth and metastasis in LLC-bearing mice. In addition, cassigarol A and piceatannol inhibited plasmin activity and the formation of capillary-like networks of human umbilical vein endothelial cells (HUVECs), suggesting that the effects of cassigarol A could be due to the inhibition of plasmin activity and formation of tubes (angiogenesis) from HUVECs [88, 89].

Viva-Natural, a natural product extracted from the dietary seaweed* Undaria pinnantifida *(Alariaceae), demonstrated therapeutic activity and moderate prophylactic activity against LLC in allogeneic mice. This product enhanced the natural cytolytic activity of peritoneal macrophages against KB cells as targets in an* in vitro* assay. The data therefore suggest that the antitumor effect of Viva-Natural may be indirect through the activation of nonspecific immune systems [90]. The antitumor potential against LLC has been evaluated in comparison with standard synthetic immunomodulators. The effect of Viva-Natural has been found to be superior to that of isoprinosine but inferior to that of MVE-2 (pyran copolymer) [91]. The combination therapy of Viva-Natural and standard anticancer drugs was additively or synergistically effective [90]. Cisplatin, 5-fluorouracil, and vincristine at low doses that were not effective when given alone, manifested antitumor activity when combined with the polysaccharide fraction of Viva-Natural, against implanted LLC (intraperitoneal,* i.p.*) in syngeneic mice. However, an additional effect on the immune system was not observed when the polysaccharide fraction of Viva-Natural was combined with the anticancer drugs at low doses. Thus, the mechanism of action of the beneficial combination is not yet clear [92].

*Brucea javanica* (Simaroubaceae) showed the anticarcinoma effect against brain metastasis as a complication of lung cancer. The results of this study showed that the median survival duration (15 months in the treatment group versus 10 months in the control group) and the quality of life of the patients in the combination of radiotherapy and intravenous (*i.v*.) injection of 10%* Brucea javanica* emulsion was much better than in the radiotherapy alone group (control). The results suggest that the* B. javanica* emulsion treatment group exhibited a synergistic action with radiotherapy in the treatment of brain metastasis as a complication of lung cancer [93]. Another study with this herbal extract showed that the preoperative* i.v.* emulsion of 10%* B. javanica* oil may improve surgical treatment of NSCLC [94].

The extract of the lichen* Cladonia leptoclada* showed significant inhibitory activity against LLC in mice. In this study, an active extract was fractionated, and the principal tumor-inhibitory constituent was characterized and identified as l-usnic acid [95].

Extracts of mistletoe (commercial sample of* Viscum album *leaf and stem) have been used for medicinal purposes for several centuries and are known to contain alkaloids with cytotoxic effects* in vitro* and* in vivo* [96]. The administration of mistletoe induced tumor growth inhibition and reduction of metastases, associated with immunomodulation. The authors reported that although lectins represent a class of active components studied of mistletoe origin, it is not likely that they alone are responsible for all effects of the plant extracts [97, 98]. A randomized phase II study of mistletoe combined with carboplatin-containing regimens was conducted in advanced NSCLC patients. No effects on quality of life or total adverse events were seen. Nevertheless, chemotherapy dose reductions, severe nonhaematological side effects, and hospitalizations were less frequent in patients treated with mistletoe, warranting further investigation as a modifier of chemotherapy-related toxicity [99]. In another study, a patient with SCLS was treated initially with extracts of mistletoe and homoeopathic treatment and appeared to respond. Subsequently, radiotherapy was given and the patient lived for five years and seven months [100]. According to the authors, the acknowledgment of the* Viscum album *extract as a phytotherapeutic adjuvant may lead to its use together with other treatments in advanced cancer biotherapy [97, 98].

Different fractions derived from the unicellular green alga* Chlorella vulgaris*, strain CK22, were tested in relation to antitumor activity against lung cancer. The most active fraction consisted of galactose-rich carbohydrate (56.1%) and protein (36.3%), suggesting that the protein moiety of the glycoprotein is mainly related to the expression of antilung cancer activity [101].

A study with* Phellinus linteus* showed that the polysaccharide fraction inhibited tumor growth in NCI-H23-implanted mice and reduced the frequency of pulmonary metastasis of B16F10 melanoma. Also studied was combination therapy with the polysaccharide fraction and Adriamycin. Although the combination was more effective in inhibiting tumor growth, this effect was not observed on the metastases [102].

Garlic (*Allium sativum*) is a common plant used mainly as a food, and it is a medicinal herb commonly used worldwide. The aged garlic extract (AGE) inhibited the growth of LL/2 lung carcinoma (syngeneic) cells transplanted into mice. AGE stimulated the proliferation of mouse spleen cells and the release of cytokines, increased NK activities, and enhanced phagocytosis by peritoneal macrophages. AGE treatment also stimulated the reactivity of lymphocytes in response to cytokines or mitogens. Therefore, it was observed that the immunostimulant effect was associated with antitumor activity [103, 104].

A clinical assay showed that treatment with* Chelidonium majus* (preparation Ukrain) in nine men with histologically proven lung cancer, previously untreated, induced an increase in the proportion of total T-cells and a significant decrease in the percentage of T-suppressor cells. The restoration of cellular immunity was accompanied by an improvement in the clinical course of the disease. This effect was particularly pronounced in patients who responded to further chemotherapy. The authors concluded that Ukrain can be immunologically effective in lung cancer patients and can improve cellular immune response [105].

The* in vivo *antitumor effect of a fraction of an ethanolic extract of* Nigella sativa* seeds was studied on LLC-implanted (*s.c*.) mice. This fraction prolonged the life span of the mice and produced significant tumor inhibition. Alpha-hederin, a triterpene saponin, was obtained from this fraction, which induced significant dose-dependent tumor inhibition, suggesting that the activity is related to the presence of this compound. However, the underlying mechanism(s) of antitumor activity of alpha-hederin remains to be established [106].

The stems and roots of* Schisandra propinqua* are a major component of the Manshanxiang Complex, a herbal medicine preparation used for the treatment of lung carcinoma in several hospitals of Yunnan Province in China [107]. In addition, the water extract from the stems and roots of* S. propinqua* shows activity against LLC in animal tests [108]. The triterpenoid manwuweizic acid, isolated from its alcoholic extract, exhibited significant inhibitory activity against LLC, suggesting that this compound is probably a major anticancer active principle of the plant [108, 109].

Review articles about the chemical properties, therapeutic benefits, and toxicity of* Withania somnifera* (ashwagandha, WS), one of the most important herbs of Ayurveda (the traditional system of medicine in India), have been published. The antitumor activity was described in different* in vitro* and* in vivo *experimental models, including urethane-induced lung adenoma in mice. The data showed that the ethanol extract of* W. somnifera *significantly reduced tumor incidence. In addition to providing protection from carcinogenic effects, the treatment also reversed the adverse effects of urethane on total leukocyte count, lymphocyte count, body weight, and mortality [110, 111].

Current studies have demonstrated that green tea polyphenols, obtained from* Camellia sinensis,* are powerful antioxidants with anticarcinogenic properties. The primary catechins in green tea are epicatechin, epicatechin-3-gallate, epigallocatechin, and epigallocatechin-3-gallate. A clinical study investigated the chemopreventive effects of green tea and coffee among cigarette smokers. The results suggest that green tea polyphenols may have an anitmutagenic effect against smoke-induced mutations in humans by blocking the cigarette-induced increase in sister-chromatid exchange frequency. It has also been shown that cultured human lung cells (A549) pretreated with green tea polyphenols and then exposed to a cigarette smoke solution or H_2_O_2_ had a reduced incidence of DNA strand breaks. Pretreatment with green tea polyphenols also reduced the overall toxicity of H_2_O_2_ as determined by cell growth after exposure. These results suggest that green tea polyphenols may inhibit DNA damage and other mutations in cells exposed to oxidants and that this effect is associated with anticarcinogenic properties [112]. A recent article published evidence from epidemiologic studies on cancer prevention by green tea. Various epidemiologic studies have examined the association between green tea consumption and lung cancer risk. The inconsistent results of those studies could be partly a result of the potential confounding effect of smoking. The data revealed that the protective effect of green tea consumption on lung cancer risk was confined to nonsmokers [113].

The effects of black tea obtained from* C. sinensis* were also studied. The administration of black tea polyphenols through the drinking water significantly inhibited 4-(methylnitrosamino)-1-(3-pyridyl)-l-butanone (NNK) and induced early bronchial cell proliferation in the short-term model. In long-term studies, administration of black tea, after a single dose of NNK, inhibited the progression of adenoma to adenocarcinoma. The cell proliferation rate in adenomas was also suppressed by black tea treatment. The authors related that such activities, at the early and late stages of lung tumorigenesis, may be important for the cancer-chemopreventive activities of black tea [114].

The antitumor activity of* Hypsizigus marmoreus*, an edible mushroom, was investigated against syngeneic tumor, LLC.* H. marmoreus* increased the life span, inhibited the activity of spontaneous tumor metastasis, and decreased the number of metastatic nodules. These effects were observed only after* i.p.* administration, but not as much by oral administration, and the data showed increased immune activity by competent cells [115].

Among the other living organisms with antitumor activity against lung cancer,* Ganoderma luncidum* (a medicinal fungus) was the most studied and has been used traditionally for the prevention and treatment of cancers for a long time in traditional Chinese medicine [37]. In this review, there were eleven reports describing the species* G. lucidum* (Ganodermataceae), where eight of them investigated the combination of* G. lucidum* extracts with antineoplastic drugs. This study showed that an aqueous extract of* G. lucidum*, called “Ling-Zhi or holy mushroom” in traditional Chinese medicine, significantly increased the life span of LLC-implanted syngeneic C57BL/6 mice, when administered* i.p.* alone or in combination with cytotoxic antitumor drugs (Adriamycin, fluorouracil, thioguanine, methotrexate, and cisplatin) or a synthetic immunomodulator (Imexon) [116]. In another study, the authors suggested that the antitumor and antimetastatic activities of the triterpenoid fraction of* G. lucidum* against LLC-implanted mice may be due to the inhibition of tumor-induced angiogenesis. The compound identified as ganoderic acid F was obtained from the acidic fraction of the triterpenoid fraction of the fruit bodies of* G. lucidum* as an active substance that inhibited the angiogenesis [117]. Recent data showed that triterpenes of* G. lucidum *have antilung cancer activity* in vitro *and* in vivo *via enhancement of immunomodulation and induction of cell apoptosis [37]. In addition,* G. tsugae*, another well-cultivated species of* Ganoderma*, was evaluated against lung cancer cells. The results revealed that the natural product inhibited the viability of H23/0.3 cells (doxorubicin-resistant lung adenocarcinoma)* in vitro *and* in vivo *and enhanced the growth-suppressive effect of doxorubicin on H23/0.3 cells. This effect is associated with the downregulation of the PI3K/Akt signaling pathway. The authors suggest that* G. tsugae* may be a useful adjuvant therapeutic agent in the treatment of lung cancer [118].

To evaluate the effect of the bee venom on the tumor growth and metastasis formation, a transplantable mammary carcinoma (MCA) was used in the syngeneic CBA mouse. The results showed that the bee venom suppressed the tumor growth and prolonged the survival of the animals compared to the controls, after* s.c. *or* i.v.* administration. However, the antimetastatic activity was observed only after* i.v.* administration, indicating that the antitumor effect of the bee venom could be highly dependent on the route of injection [119].

Considering all the countries covered in the present study, China, Japan, South Korea, and Ethiopia were those that published the most studies of species with antitumor activity against lung cancer: ten works from China, eight works from Japan, and four publications from South Korea and Ethiopia each. The most used cell model for the investigation of antitumor activity against lung cancer was LLC. Of the 58 studies that showed this cell line as the experimental model used, 44 showed its sensitivity to the samples tested. Those data indicated that this cell line is the most used model worldwide to evaluate the antitumor activity in lung cancer cells. Other models cited in few studies included NSCLC and adenomas.

## 3. Conclusions

Several countries have studied medicinal plants with antitumor potential, including against lung cancer. Because many species are active in different experimental models, the natural products from plants continue to be a rich source of herbal medicines or biologically active compounds. There is a need for further studies on the standardization or chemical characterization of the extracts used. With respect to pharmacological studies, most of them were performed in mice. However, there were also studies with humans for various species of plants. This review shows that many medicinal herbs have been examined for antilung cancer activity, presenting important results that provide insight into their use for the treatment of cancer. However, despite many studies showing the evaluation of possible mechanisms of action of these compounds, the majority of studies still presented only preliminary screening data and therefore did not describe any mechanism of action. For these studies, they are classified only as “active.” In addition, new research findings have shown extracts as potential phytotherapeutic adjuvants in advanced lung cancer therapy. This could lead to greater safety and benefits for people, contributing to a better access to health care and thereby a better quality of life of patients with lung cancer.

## Figures and Tables

**Table 1:** Medicinal plants and other organisms with antitumor potential against lung cancer.

Family and botanical name	Origin	Part used	Extract	Model	Mammal tested	Result	Reference
Agavaceae							
*Yucca aloifolia *	USA	Flowers	MeOH extract	Ca-lewis lung	Mouse	Active	[120]

Alariaceae							
*Undaria pinnatifida *	Hawaii	Commercial sample of thallus pacific	Type of extract not stated	Ca-lewis lung	Mouse	Active	[90]
	Hawaii	Commercial sample of thallus pacific	H_2_O-insoluble extract	Ca-lewis lung	Mouse	Active	[90]
	Japan	Commercial sample of thallus pacific	H_2_O Extract	Ca-lewis lung	Mouse	Active	[91]
	Japan	Dried thallus pacific	Polysaccharide fraction	Ca-lewis lung	Mouse	Active	[92]

Anacardiaceae							
*Semecarpus anacardium *	India	Dried fruit	CHCl_3_ extract	Ca-lewis lung	Mouse	Inactive	[121]

Apidae							
*Apis mellifera *	Croatia	Fresh venom	Venom	Decreased number of lung metastases	Mouse	Active	[119]

Apocynaceae							
*Ervatamia heyneana *	India	Leaf	MeOH extract	Ca-lewis lung	Mouse	Inactive	[122]

ARALIACEAE							
*Eleutherococcus senticosus *	USSR	Root	EtOH (16%) extract	Chemical induced tumor	Mouse	Active	[123]
*Panax ginseng *	South Korea	Dried root	H_2_O extract	Mice exposed to benzopyrene	Mouse	Active	[86]
	South Korea	Dried root	H_2_O extract	Aflatoxin-induced lung adenoma	Mouse	Active	[124]
	South Korea	Dried root	H_2_O extract	Fluorenyl-induced lung adenoma	Mouse	Inactive	[124]
	South Korea	Dried root	H_2_O extract	DMBA-induced lung adenoma	Mouse	Inactive	[124]
	South Korea	Dried root	H_2_O extract	Urethane-induced lung adenoma	Mouse	Active	[124]

Arecaceae							
*Calamus rotang *	India	Aerial parts	EtOH-H_2_O (50%) extract	Ca-lewis lung	Mouse	Active	[125]

Asteraceae							
*Tagetes minuta *	Ethiopia	Aerial parts	H_2_O extract	Ca-lewis lung	Mouse	Active	[126]

Celastraceae							
*Maytenus serrata *	Ethiopia	Fruit	EtOH (95%) extract	Ca-lewis lung	Mouse	Active	[127]
	Ethiopia	Stemwood	EtOH (95%) extract	Ca-lewis lung	Mouse	Active	[127]
	Ethiopia	Root	EtOH (95%) extract	Ca-lewis lung	Mouse	Active	[127]
	Kenya	Root	EtOH (95%) extract	Ca-lewis lung	Mouse	Active	[127]
	Kenya	Stemwood	EtOH (95%) extract	Ca-lewis lung	Mouse	Active	[127]

Chlorellaceae							
*Chlorella vulgaris *	Pacific (Japan)	Dried cells	Chromatographic fraction	Ca-lung-3LL	Mouse	Active	[101]

Cladoniaceae							
*Cladonia leptoclada *	New Zealand	Thallus	EtOH-H_2_O (1 : 1) extract	Ca-lewis lung	Mouse	Active	[95]

Euphorbiaceae							
*Croton macrostachys *	Ethiopia	Fruit	EtOH (95%) extract	Ca-lewis lung	Mouse	Active	[87]
*Euphorbia esula *	USA	Aerial parts	EtOH (95%) extract	Ca-lewis lung	Mouse	Inactive	[128]
	USA	Entire plant	EtOH (95%) extract	Ca-lewis lung	Mouse	Active	[129]
*Euphorbia fischeriana *	China	Dried entire plant	EtOH (95%) extract	Ca-lewis lung	Mouse	Active	[130]
*Euphorbia ingens *	South Africa	Fresh stem	EtOH-H_2_O (1 : 1) extract	Ca-lewis lung	Mouse	Inactive	[131]
*Jatropha gossypiifolia *	Costa Rica	Root	H_2_O extract	Ca-Lewis lung	Mouse	Inactive	[132]
	Costa Rica	Root	EtOH (95%) extract	Ca-Lewis lung	Mouse	Inactive	[133]

Fabaceae							
*Cassia garrettiana *	Thailand	Dried heartwood	MeOH extract	Ca-lewis lung	Mouse	Active	[88]
	Thailand	Dried heartwood	MeOH extract	Ca-lewis lung	Mouse	Active	[89]
*Sophora flavescens *	China	Dried root	Type of extract not stated	Ca-lewis lung	Mouse	Active	[134]
*Sutherlandia frutescens *	South Africa	Fresh flowers	EtOH-H_2_O (1 : 1) extract	Ca-lewis lung	Mouse	Inactive	[131]

Ganodermataceae							
*Ganoderma lucidum *	Taiwan	Dried fruit body	H_2_O extract	Ca-lewis lung	Mouse	Active	[116]
	Taiwan	Dried fruit body	EtOH (95%) extract	Ca-lewis lung	Mouse	Active	[116]
	Taiwan	Dried fruit body	H_2_O extract in combination with adriamycin	Ca-lewis lung	Mouse	Active	[116]
	Taiwan	Dried fruit body	H_2_O extract in combination with cisplatin	Ca-lewis lung	Mouse	Active	[116]
	Taiwan	Dried fruit body	H_2_O extract in combination with fluorouracil	Ca-lewis lung	Mouse	Active	[116]
	Taiwan	Dried fruit body	H_2_O extract in combination with methotrexate	Ca-lewis lung	Mouse	Active	[116]
	Taiwan	Dried fruit body	H_2_O extract in combination with imexon	Ca-lewis lung	Mouse	Active	[116]
	Taiwan	Dried fruit body	H_2_O extract in combination with thioguanine	Ca-lewis lung	Mouse	Active	[116]
	Taiwan	Dried mycelium	Hot H_2_O extract	Ca-lewis lung	Mouse	Active	[135]
	South Korea	Myceliu	Hot H_2_O extract	Ca-lewis lung	Mouse	Active	[136]
	Japan	Dried fruit body	H_2_O extract	Ca-lewis lung	Mouse	Active	[117]

Geraniaceae							
*Pelargonium graveolens *	China	Dried root	Type of extractnot stated	Ca-lewis lung	Mouse	Active	[134]

Hymenochaetaceae							
*Phellinus linteus *	South Korea	Mycelium	Polysaccharide fraction	Cancer cell line NCI-H23	Mouse	Active	[102]

Liliaceae							
*Allium sativum *	Japan	Aged bulb	Aged garlic extract used	Sarcoma-180 and LL/2 lung carcinoma cells	Mouse	Active	[103, 104]

Loranthaceae							
*Viscum album *	Germany	Dried aerial parts	H_2_O soluble fraction	Ca-lewis lung	Mouse	Inactive	[137]
	England	Commercial sample of leaf + stem	Type of extract not stated	Small cell lung	Human adult	Active	[100]
	Switzerland	Commercial sample of entire plan	H_2_O Ext	Ca-lewis lung	Mouse	Active	[96]

Malvaceae							
*Thespesia populnea *	India	Fruit	EtOH-H_2_O (1 : 1) extract	Ca-lewis lung	Mouse	Active	[125]
*Hibiscus syriacus *	Taiwan	Dried root bark	Acetone extract	Ca-human lung	Mouse	Inactive	[138]

Melastomataceae							
*Memecylon umbellatum *	India	Leaf	EtOH-H_2_O (1 : 1) extract	Ca-lewis lung	Mouse	Inactive	[125]

Nyctaginaceae							
*Mirabilis multiflora *	USA	Root	H_2_O extract	Ca-lewis lung	Mouse	Inactive	[139]

Nyssaceae							
*Nyssa sylvatica *	Not stated	Stem bark	H_2_O extract	Ca-lewis lung	Mouse	Inactive	[140]

Ochnaceae							
*Lophira lanceolata *	Nigeria	Root bark	EtOH-H_2_O (1 : 1) extract	Ca-lewis lung	Mouse	Inactive	[141]

Oscillatoriaceae							
*Oscillatoria acutissima *strain B.1	Hawaii	Freeze-dried organism	Type of extract not stated	Ca-lewis lung	Mouse	Inactive	[142]

Papaveraceae							
*Chelidonium majus *	Austria	Dried entire plant	Alkaloid fraction	Lung cancer	Human adult	Active	[105]

Pectinidae							
*Patinopecten yessoensis *	Pacific (Japan)	Fresh organism	Polysaccharide fraction	Cells human embryonic lung	Mouse	Inactive	[143]

Ranunculaceae							
*Nigella sativa *	India	Seed	EtOH-H_2_O (50%) extract	Ca-lewis lung	Mouse	Active	[125]
	Singapore	Commercial sample of seed	Chromatographic fraction	Ca-lewis lung	Mouse	Active	[106]

Rubiaceae							
*Morinda citrifolia *	Hawaii	Dried fruit juice	EtOH insoluble fraction	Ca-lewis lung	Mouse	Active	[144]

Rhizophoraceae							
*Bruguiera sexangula *	Papua-New Guinea	Stem bark	EtOH (95%) extract	Ca-lewis lung	Mouse	Active	[145]

Schisandraceae							
*Schisandra propinqua *	China	Dried root + stem	Hot H_2_O extract	Ca-human lung	Human adult	Active	[109]

Simaroubaceae							
*Brucea javanica *	China	Dried part not specified	Type of extract not stated	Lung cancer	Human adult	Active	[93]
	China	Seed	Seed oil	Squamous carcinoma and adenocarcinoma	Human adult	Active	[94]
	China	Seed	Seed oil	The extract showed a therapeutic effect for lung cancers	Human adult	Active	[146]
	China	Seed	Fixed oil	Patients with lung cancer	Human adult	Active	[147]

Solanaceae							
*Withania somnifera *	India	Dried root	EtOH (95%) extract	Lung adenomas	Mouse	Active	[110]
	India	Dried entire plant	EtOH (95%) extract	Lung adenomas	Mouse	Active	[148]

Theaceae							
*Camellia sinensis *	China	Dried leaf	Polyphenolic fraction	Lung cancer	Mouse	Active	[149]
	China	Green leaf	Tea	Lung cancer	Human adult	Active	[112]
	China	Black leaf	Leaves	Reduced tumor multiplicity and volume in nnk treated mice	Mouse	Active	[114]

Tricholomataceae							
*Hypsizygus marmoreus *	Japan	Dried fruit	H_2_O extract	Ca-lewis lung	Mouse	Active	[115]
*Lentinus edodes *	Japan	Dried fruit body	H_2_O extract	Ca-lewis lung	Mouse	Inactive	[150]

Violaceae							
*Viola odorata *	South Africa	Fresh leaf	EtOH-H_2_O (1 : 1) extract	Ca-lewis lung	Mouse	Inactive	[131]
